# Molecular subtypes of breast cancer are associated with characteristic DNA methylation patterns

**DOI:** 10.1186/bcr2590

**Published:** 2010-06-18

**Authors:** Karolina Holm, Cecilia Hegardt, Johan Staaf, Johan Vallon-Christersson, Göran Jönsson, Håkan Olsson, Åke Borg, Markus Ringnér

**Affiliations:** 1 Department of Oncology, Clinical Sciences, Lund University, Barngatan 2B, SE-221 85 Lund, Sweden; 2 CREATE Health Strategic Centre for Translational Cancer Research, Lund University, BMC C13, SE-221 84 Lund, Sweden; 3 Lund Strategic Research Center for Stem Cell Biology and Cell Therapy, Lund University, BMC B10, SE-221 84 Lund, Sweden

## Abstract

**Introduction:**

Five different molecular subtypes of breast cancer have been identified through gene expression profiling. Each subtype has a characteristic expression pattern suggested to partly depend on cellular origin. We aimed to investigate whether the molecular subtypes also display distinct methylation profiles.

**Methods:**

We analysed methylation status of 807 cancer-related genes in 189 fresh frozen primary breast tumours and four normal breast tissue samples using an array-based methylation assay.

**Results:**

Unsupervised analysis revealed three groups of breast cancer with characteristic methylation patterns. The three groups were associated with the luminal A, luminal B and basal-like molecular subtypes of breast cancer, respectively, whereas cancers of the HER2-enriched and normal-like subtypes were distributed among the three groups. The methylation frequencies were significantly different between subtypes, with luminal B and basal-like tumours being most and least frequently methylated, respectively. Moreover, targets of the polycomb repressor complex in breast cancer and embryonic stem cells were more methylated in luminal B tumours than in other tumours. *BRCA2*-mutated tumours had a particularly high degree of methylation. Finally, by utilizing gene expression data, we observed that a large fraction of genes reported as having subtype-specific expression patterns might be regulated through methylation.

**Conclusions:**

We have found that breast cancers of the basal-like, luminal A and luminal B molecular subtypes harbour specific methylation profiles. Our results suggest that methylation may play an important role in the development of breast cancers.

## Introduction

Breast cancer is a complex and heterogeneous disease and one of the leading causes of death among women. Tumourigenesis is a multistep process resulting from the accumulation of genetic alterations such as mutations, rearrangements and copy number variations, but also epigenetic alterations such as promoter methylation and histone modification [1,2]. DNA methylation plays an essential role in development, chromosomal stability, and for maintaining gene expression states [1]. DNA methylation occurs when methyl groups are added to cytosines in CpG dinucleotides, leading to a closed chromatin conformation and gene silencing. CpGs are often found at increased frequencies in promoter regions, forming CpG islands. Hypermethylation of CpG islands affects genes involved in cell cycle control, DNA repair, cell adhesion, signal transduction, apoptosis and cell differentiation [1-3]. In tumour cells, local promoter hypermethylation is often accompanied by global hypomethylation [1]. This results in more global patterns of methylation as compared with mutation spectra, which differ greatly in extent and patterns between tumours [4].

Gene silencing and maintenance of cellular identity can also be mediated by histone modifications carried out by polycomb group (PcG) proteins. Enhancer of zeste homolog 2 (EZH2) is a core member of the polycomb repressive complex 2 (PRC2) that catalyses the histone mark characteristic for PcG-mediated silencing: trimethylation of lysine 27 on histone H3 (H3K27me3), which leads to the blocking of transcriptional activation factors and thereby gene silencing independent of promoter methylation [5]. Other members of the PRC2 complex include suppressor of zeste 12 homolog (SUZ12) and embryonic ectoderm development (EED) [6]. PRC2 target genes are involved in embryonic development, differentiation and cell fate decisions [7]. PcG proteins are thought to silence genes in a very dynamic fashion [8]. In cancer cells, the presence of PRC2 can lead to recruitment of DNA methyltransferases (DNMTs) resulting in *de novo *DNA methylation and more permanent repression of PRC2 target genes [9]. Moreover, many of the genes that undergo promoter methylation in cancer are already expressed at low levels in corresponding normal cells, suggesting that a large fraction of *de novo *methylation events in cancer cells are not subject to growth selection but instead reflect an instructive mechanism inherent of the normal cell from which the tumour originated [10,11].

Several microarray studies have shown that breast tumours can be divided into at least five molecular subtypes based on gene expression profiles [12-14]. These subtypes (basal-like, luminal A (lumA), luminal B (lumB), human epidermal growth factor receptor 2 (HER2)-enriched and normal-like) have been suggested to originate from different precursor cells and follow different progression pathways. Herein, we investigated whether the molecular subtypes show specific methylation patterns by analysing a panel of 807 cancer-related genes in 189 breast tumours. We report that the breast cancer subtypes, especially lumA, lumB and basal-like, demonstrate different methylation profiles.

## Materials and methods

### Patients and tumours

Fresh frozen primary tumour tissue from 189 breast cancer patients, including 15 *BRCA1 *and 13 *BRCA2 *mutation carriers, 43 non-*BRCA1/2*-familial (familial), 115 sporadic and 3 cases with unknown family status, were obtained from the Southern Sweden Breast Cancer Group's tissue bank at the Department of Oncology at Skåne University Hospital in Sweden. All tumours were macrodissected and evaluated for tumour cell content by an experienced pathologist. Moreover, the majority (168/189) of samples were analysed by array comparative genomic hybridization (aCGH) and found to display genomic profiles with aberrations consistent with the presence of a large fraction of tumour cells. Normal breast tissue from four breast cancer patients was also included. Patient and tumour characteristics are shown in Table 1. The study was approved by the regional ethical committee at Lund University (reg. no. LU240-01 and 2009/658), waiving the requirement for informed consent for the study.

**Table 1:** Patient and tumour characteristics for the 189 patients

Characteristic	Basal-like (n = 43) (%)	LumA (n = 46) (%)	LumB (n = 35) (%)	HER2-enriched (n = 14) (%)	Normal-like (n = 17) (%)	Non-classified (n = 24) (%)	Non-GEX (n = 10) (%)	Total (n = 189) (%)
Family status								
BRCA1	9 (21)	1 (2)	1 (3)	0	1 (6)	0	3 (43)	15 (8)
BRCA2	1 (2)	1 (2)	7 (20)	0	0	0	4 (57)	13 (7)
Familial	7 (16)	10 (22)	11 (31)	3 (21)	5 (29)	7 (29)	0	43 (23)
Sporadic	26 (60)	34 (74)	16 (46)	11 (79)	11 (65)	17 (71)	0	115 (62)
Unknown	0	0	0	0	0	0	3	3
ER status								
Positive	2 (5)	44 (96)	31 (91)	5 (36)	10 (67)	20 (83)	1 (100)	113 (65)
Negative	38 (95)	2 (4)	3 (9)	9 (64)	5 (33)	4 (17)	0	61 (35)
Unknown	3	0	1	0	2	0	9	15
PgR status								
Positive	2 (5)	44 (96)	28 (82)	5 (36)	8 (53)	20 (83)	1 (100)	108 (62)
Negative	38 (95)	2 (4)	6 (18)	9 (64)	7 (47)	4 (17)	0	66 (38)
Unknown	3	0	1	0	2	0	9	15
Histological grade								
Grade 1	0	9 (28)	3 (10)	0	1 (8)	5 (23)	0	18 (12)
Grade 2	2 (5)	20 (63)	10 (34)	3 (27)	8 (62)	5 (23)	0	48 (32)
Grade 3	39 (95)	3 (9)	16 (55)	8 (73)	4 (31)	12 (54)	0	82 (55)
Unknown	2	14	6	3	4	2	10	41
Node status								
Negative	28 (68)	36 (80)	17 (61)	11 (85)	9 (56)	16 (70)	0	117 (70)
Positive	13 (32)	9 (20)	11 (39)	2 (15)	7 (44)	7 (30)	0	49 (30)
Unknown	2	1	7	1	1	1	10	23
Age (median)	46	49.5	48	45.5	49	48.5	na	48

Cases for which data are unknown are excluded from total when calculating percentage.

ER, oestrogen receptor; GEX, gene expression; HER2, human epidermal growth factor receptor 2; lumA, luminal A; lumB, luminal B; na, not available; PgR, progesterone receptor.

### DNA isolation

Genomic DNA was isolated from fresh frozen primary breast tumours in a three-step procedure. Tumour cells were pre-treated with proteinase K (20 mg/ml) at 55°C over-night, DNA was purified using the Promega Wizard Genomic DNA Purification kit (Promega Corporation, Madison, WI, USA) and finally DNA was further purified by phenol/chloroform treatment in phase-lock tubes. DNA was quantified using a NanoDrop (ThermoScientific, Wilmington, DE, USA).

### Methylation analysis

Bisulfite conversion of 500 ng genomic DNA was performed using the EZ DNA Methylation kit (Zymo Research, Orange, CA, USA) following the manufacturer's protocols. Methylation analysis was performed using Illumina GoldenGate Methylation Cancer Panel I (Illumina, San Diego, CA, USA) [15]. In this panel 1,505 CpG loci corresponding to 807 cancer-related genes are analysed simultaneously. Primers designed to match either the methylated or unmethylated state of a CpG site are hybridised to bisulfite-converted DNA. After an extension and ligation step the templates are amplified using two different fluorescently labelled universal primers, one for each methylation state, and then hybridised to corresponding sequences on an array. For each CpG site, methylation status is essentially calculated as the ratio of fluorescence from the methylated state over the sum of fluorescence from the methylated and unmethylated states, and presented as a β-value [15]. The β-values are continuous values between 0 and 1, with 0 corresponding to completely unmethylated sites and 1 to completely methylated sites. The methylation data have been deposited in NCBI's Gene Expression Omnibus (GEO) [16,17] and are accessible through GEO Series accession number [GEO:GSE22210].

### Gene expression and DNA copy number data sets

The majority of the tumours (179/189) are part of a larger set (n = 577) with gene expression data obtained using oligonucleotide arrays (GEO Platform GPL5345) produced at the SCIBLU Genomics Centre at Lund University, Sweden [18] as described by Jönsson *et al. *[19] and processed as described [20]. Briefly, expression levels have been centred across all 577 samples to obtain expression levels relative to a large set of breast tumours. Also, samples have been classified into molecular subtypes according to the gene expression centroids published by Hu *et al. *[14] as described [21], with samples having Pearson correlations smaller than 0.2 to all centroids considered to be non-classified. Relative expression levels for all 511 oligonucleotide probes for genes with CpG sites on our methylation assays are available in Additional File 1. For analysis of expression of *EZH2 *and PRC2 targets, we used all 286 (of 577) tumours that were primary tumours, Swedish, and classified into a subtype [see Additional File 2]. For 168 of 189 tumours, aCGH data were available as part of another study [20]. For aCGH, BAC arrays with more than 32,000 clones (GEO Platform GPL4723) were produced at the SCIBLU Genomics Centre at Lund University, Sweden [18] as described [19], and analysed as described [22]. Gain of *EZH2 *and the fraction of genome altered were calculated as described [23] [see Additional File 3].

### Data analysis

The Beadstudio Methylation Module (Illumina, San Diego, CA, USA) was used for data extraction, normalisation and quality control. β-values for all 1,452 CpG sites (corresponding to 803 genes) that passed Beadstudio quality control are available for all 189 tumours and 4 normal samples [see Additional File 4]. β-values were stratified into three groups, all values 0.3 or below were set to 0, values above 0.3 and below 0.7 were set to 0.5, and finally values 0.7 and above were set to 1 and interpreted as hypermethylated. Methylation frequencies for samples were calculated as the fraction of CpGs with value 1. Stratified data were used for all subsequent analyses. Stratified β-values were mean-centred across all tumours to generate relative methylation levels. Relative methylation levels for all 189 tumours and 1,452 CpG sites are available in Additional File 5.

Clustering analyses were performed in MultiExperiment Viewer (MeV) [24] using relative methylation levels and the most variable CpG sites by excluding those with a standard deviation less than 0.3 across samples. Hierarchical clustering was performed using Pearson correlation distance and average linkage. K-means clustering was performed using Pearson correlation distance. Associations between subtypes and clusters were assessed using Fisher's exact test in R [25] on 2 × 2 contingency tables for the 179 tumours with expression data. Differentially methylated CpGs were identified using analysis of variance (ANOVA) with five groups, 1,000 permutations, and a false significant number of 10 or less (corresponding to false discovery rate (FDR) < 1%) in MeV. Significance analysis of microarrays (SAM) [26] with 1,000 permutations and FDR of 0% was used in MeV to identify significant CpGs for each subtype, using two-class comparisons between tumour samples belonging to a subtype and all other tumour samples. Survival analysis was performed in R using the survival package. For each CpG site, the correlation between expression and methylation was calculated and the global association was assessed using a binomial test for the number of negative correlation coefficients. Fisher's exact test, binomial test, t-test, ANOVA and Wilcoxon test were performed in R. All tests were two-sided.

Following Ben-Porath *et al. *[27] we used a gene set for PRC2 targets consisting of the 654 genes identified by Lee *et al. *[28] using chromatin immunoprecipitation (ChIP) arrays as bound by all of SUZ12, EED, and H3K27me3 in human embryonic stem (ES) cells. To explore genes under PRC2 control in breast tumour cells, we used three gene sets: (i) 853 genes identified by Gupta *et al. *[29] using ChIP arrays as being occupied by EZH2, SUZ12 and H3K27me3 after *HOTAIR *overexpression in the oestrogen receptor (ER)-negative breast cancer cell line MDA-MB-231, (ii) the top 600 promoters (mapped to 451 genes) identified by Squazzo *et al. *[30] using ChIP arrays as being occupied by SUZ12 in the ER-positive breast cancer cell line MCF7, and (iii) 44 genes identified by Tan *et al. *[31] using RNA interference, expression arrays and ChIP studies as being selectively repressed by PRC2 in MCF7. For each tumour, we calculated the average relative methylation of a gene set as the average of the relative methylation levels of all CpG sites matching a gene in the gene set. Similarly, we calculated the average relative expression of a gene set as the average of the expression levels for all genes in the gene set.

## Results

### Unsupervised clustering reveals molecular subtype-specific methylation patterns

**Figure 1:**
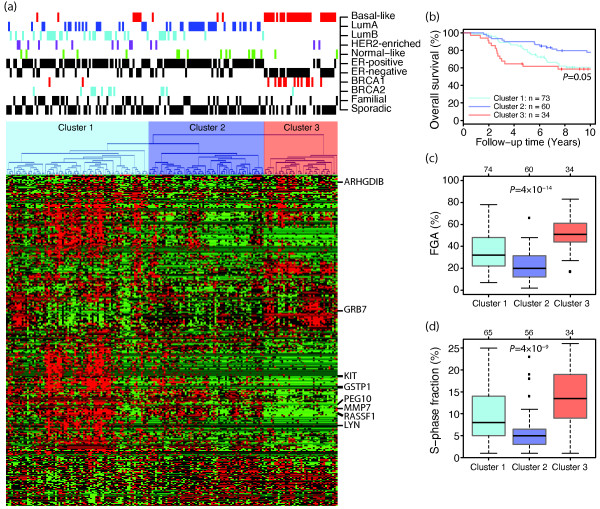
**Unsupervised clustering of 189 tumours based on the 332 most variably methylated CpGs**. **(a) **Hierarchical clustering. The heatmap shows relative methylation levels (red, more methylated; green, less methylated). Clustering results in three clusters associated with lumB, lumA and basal-like tumours, respectively. **(b) **Kaplan-Meier demonstrating longest survival in lumA-associated Cluster 2 and shortest in basal-like-associated Cluster 3. *P*-value was calculated using log-rank test. **(c) **Fraction of genome altered (FGA) highest in basal-like-associated Cluster 3 and lowest in lumA-associated Cluster 2. *P*-value was calculated using analysis of variance (ANOVA). **(d) **S-phase fraction highest in basal-like-associated Cluster 3 and lowest in lumA-associated Cluster 2. *P*-value was calculated using ANOVA. The number of tumours in each subtype is shown at top.

Hypermethylation was observed in all 189 breast tumours, on average affecting 31% of all analysed CpG sites. Unsupervised hierarchical clustering using the 332 most variably methylated CpG loci, corresponding to 247 genes, divided the tumours into three main branches (Figure 1a) [see Additional File 6]. The division into the two main branches is mainly dependent on ER status (*P *= 2 × 10^-13^, Fisher's exact test). The branch with predominantly ER-negative tumours is associated with the basal-like subtype (*P *= 6 × 10^-22^). The second division splits the predominantly ER-positive luminal tumours into two clusters, one associated with lumA tumours (*P *= 0.0004), and another containing a mixture of all subtypes, but including the majority of lumB (*P *= 0.0002) and HER2-enriched (*P *= 0.03) tumours. Normal-like tumours are found in all clusters. Survival analysis demonstrated expected results with best outcome in the lumA-associated cluster and worst outcome in the basal-like-associated cluster (*P *= 0.05, log-rank test; Figure 1b) [13,14]. Additionally, for the samples with aCGH data (169/189) we investigated the fractions of the genome altered, representing the percentage of BAC clones subjected to gain or loss for each sample. We found larger fractions altered in tumours in the basal-like-associated Cluster 3 and smaller fractions in tumours of the lumA-associated Cluster 2 (*P *= 4 × 10^-14^, ANOVA; Figure 1c) corroborating earlier findings by Hu *et al. *[32]. We used S-phase fraction as a measure of cellular proliferation of tumours to further delineate the differences between the clusters. The clusters contained tumours with significantly different S-phase fractions (*P *= 4 × 10^-9^, ANOVA; Figure 1d). As expected, tumours in the basal-like-associated cluster had the highest S-phase fractions, and tumours in the lumB-associated cluster had higher S-phase fractions than tumours in the lumA-associated cluster.

To investigate the robustness of the results from the hierarchical clustering, K-means clustering was performed varying the number of clusters (K) from two to five [see Additional File 7]. For a K of 2, we found one cluster associated with lumA (*P *= 6 × 10^-4^, Fisher's exact test) and lumB (*P *= 6 × 10^-9^) tumours and one cluster associated with basal-like tumours (*P *= 5 × 10^-14^). For a K of 3, we found, as for the hierarchical clustering, that the three clusters were significantly associated with lumA (*P *= 3 × 10^-6^), lumB (*P *= 4 × 10^-5^) and basal-like (*P *= 2 × 10^-26^) tumours, respectively. For a K of 4, we again found three clusters associated with lumA (*P *= 2 × 10^-6^), lumB (*P *= 2 × 10^-6^) and basal-like (*P *= 3 × 10^-20^) tumours, respectively, whereas the remaining cluster was the smallest (11% of tumours) and contained a mixture of subtypes. For a K of 5, two clusters were associated with lumA tumours (*P *= 0.001 and *P *= 0.01, respectively), one cluster with lumB tumours (*P *= 1 × 10^-7^), one cluster with basal-like tumours (*P *= 8 × 10^-25^), whereas the remaining cluster again was the smallest (12% of tumours) and contained a mixture of subtypes. For smaller K, normal-like tumours were found in most clusters, but for a K of 5, 13 of 17 normal-like tumours were in the two lumA-associated clusters (*P *= 0.01). However, HER2-enriched tumours were for all K present in all clusters. In summary, based on the investigated panel of CpGs, the methylation pattern of basal-like tumours clearly differs from that of other subtypes and a group dominated by lumB tumours appears to be more frequently methylated (Figure 1a).

### Array-based methylation analysis corroborates individual CpG sites associated with clinical parameters

To validate the performance of our methylation assay, we investigated the relative methylation levels of genes previously reported as having methylation patterns associated with ER and HER2 status in breast tumours. Sunami *et al. *investigated methylation of eight tumour-related genes in breast tumours using methylation-specific PCR and capillary-array electrophoresis analysis, and identified *RASSF1*, *GSTP1 *and *APC *as having significantly lower methylation frequencies in tumours that were ER-negative and HER2-negative (double negative) compared with tumours that were either ER-positive or HER2-positive [33]. Seven CpG sites for these three genes were present on our array (Table 2). In concordance with the results by Sunami *et al*., we found the methylation levels of all these seven CpG sites to be significantly lower in the basal-like subtype (corresponding to their group of double-negative tumours) compared with the luminal or HER2-enriched subtypes (corresponding to their ER-positive or HER2-positive tumours; Table 2). Moreover, we found all CpG sites for *RASSF1 *and *APC*, but none for *GSTP1*, to have significantly higher methylation levels in ER-positive than in ER-negative tumours (Table 2). We conclude that our assay recapitulates findings by others using a different method in independent tumours. Also, the accuracy and reproducibility of the platform have been thoroughly validated elsewhere [15,34,35].

**Table 2:** Average relative methylation levels of genes previously associated with ER and HER2 status

CpG site	Basal-like (n = 43)	Luminal or HER2-enriched (n = 95)	P-value^1^	ER-negative (n = 61)	ER-positive (n = 113)	*P*-value^1^
RASSF1_E116_F	-0.37	0.16	2 × 10^-11^	-0.22	0.13	2 × 10^-7^
RASSF1_P244_F	-0.32	0.16	2 × 10^-11^	-0.19	0.12	3 × 10^-7^
GSTP1_E322_R	-0.24	0.07	2 × 10^-5^	-0.02	0.03	0.4
GSTP1_P74_F	-0.11	0.03	0.02	-0.04	0.03	0.2
GSTP1_seq_38_S153_R	-0.10	0.05	6 × 10^-3^	-0.01	0.02	0.7
APC_P14_F	-0.38	0.11	2 × 10^-8^	-0.19	0.11	5 × 10^-5^
APC_P280_R	-0.13	0.07	2 × 10^-4^	-0.10	0.05	5 × 10^-4^

^1^Wilcoxon test.

ER, oestrogen receptor; HER2, human epidermal growth factor receptor 2

### Methylation status correlates with gene expression

Next, we studied correlations between methylation status and gene expression. All CpG sites for which we had methylation data were matched based on gene symbols to available gene expression data, and methylation levels were correlated with gene expression levels across tumour samples. This approach identified 470 unique genes represented by 832 CpG sites and by 511 oligonucleotide probes on the expression arrays. In total there were 906 pairs of CpG sites and oligonucleotide probes with the same gene symbol for both platforms [see Additional File 8]. For 113 of these 906 methylation-expression pairs, the relative methylation level of the CpG site did not change across the tumours. A highly significant fraction (569 pairs, 72%) of the remaining 793 expression-methylation pairs with varying relative methylation levels showed inverse correlation between relative methylation levels and expression levels (*P *= 2 × 10^-35^, binomial test). Thus, we found an inverse correlation between methylation and gene expression for a similar fraction of CpG sites as has previously been found for follicular lymphoma using the same methylation assay [35].

### High methylation frequency among luminal B tumours

**Figure 2:**
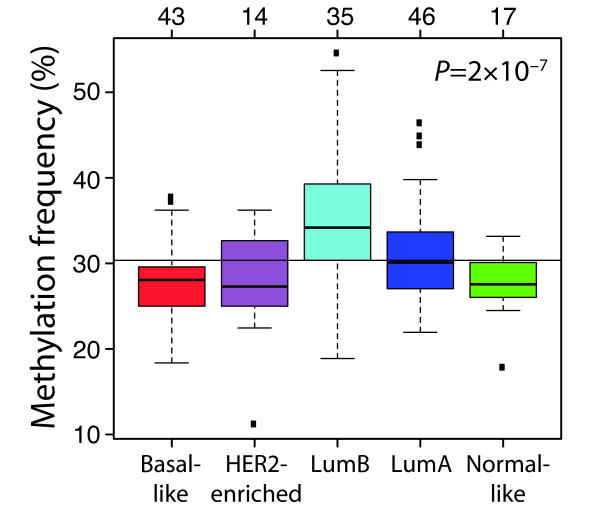
**Boxplot stratified by subtype for methylation frequencies of the 196 subtype-associated CpGs**. These CpGs are more frequently methylated in lumB tumours and less methylated in basal-like tumours. *P*-value was calculated using analysis of variance. The number of tumours in each subtype is shown at top.

To further study variations in methylation frequencies we used ANOVA to identify 196 CpGs (corresponding to 163 genes) with methylation patterns associated with the molecular subtypes [see Additional File 9]. Methylation frequencies for these CpGs were calculated for molecular subtype, family status, hormone receptor status, histological grade, node status, age, tumour size and tissue (Table 3). The methylation frequency of these CpGs was significantly different between the molecular subtypes (*P *= 2 × 10^-7^, ANOVA). The CpGs were in particular found to be more frequently methylated in lumB tumours and less methylated in basal-like tumours (Figure 2). Comparing tumours based on ER status, irrespective of molecular subtype, a higher methylation frequency was observed in ER-positive and progesterone receptor (PgR)-positive tumours (*P *= 0.005 and *P *= 0.02, respectively, t-test). Tumours from germline *BRCA2 *mutation carriers had a higher degree of CpG methylation as compared with *BRCA1*-mutated, other familial and sporadic tumours (*P *= 0.007, ANOVA). Additionally, the average methylation frequency of the subtype-associated CpGs was lower in normal breast tissue than in tumours (*P *= 2 × 10^-4^, t-test). However, stratifying the tumours by molecular subtype, significantly lower average methylation frequency in normal breast tissue was only found when comparing with lumA (*P *= 2 × 10^-4^, t-test) and lumB (*P *= 2 × 10^-6^) tumours, respectively.

**Table 3:** Average methylation frequency for the 196 subtype-associated CpGs

	Methylation frequency (%)		Number of patients	*P*-value^1^
			
	Average	SD		
Molecular subtype^2^				**2 × 10^-7^**
Basal-like	27.6	4.1	43	
LumA	31.1	5.5	46	
LumB	35.1	7.9	35	
HER2-enriched	27.8	6.4	14	
Normal-like	27.5	3.6	17	
Non-classified	29.9	6.4	24	
Non-GEX	34.3	8.7	10	
Family status				**0.007**
BRCA1	29.8	7.4	15	
BRCA2	36.5	8.3	13	
Familial	30.3	6.6	43	
Sporadic	29.9	6.0	115	
ER status^3^				**0.005**
Positive	31.3	7.0	113	
Negative	28.6	5.4	61	
PgR status^3^				**0.02**
Positive	31.3	6.8	108	
Negative	28.9	6.0	66	
Histological grade				0.7
Grade 1	29.6	6.7	18	
Grade 2	30.9	6.2	48	
Grade 3	29.7	7.0	82	
Node status				0.7
Positive	29.8	6.5	49	
Negative	30.3	6.4	117	
Age (years)				0.5
< 50	30.0	6.4	108	
≥ 50	30.7	6.4	71	
Size (mm)				0.3
≤ 20	30.6	6.4	90	
> 20	28.7	6.3	76	
Tissue				**2 × 10^-4^**
Normal breast	27.3	0.9	4	
Tumour	30.5	6.6	189	

^1^t-test for two categories, otherwise one-way analysis of variance. *P*-values < 0.05 in bold.

^2^*P*-value between subtypes basal-like, lumA, lumB, HER2-enriched and normal-like.

^3^Tumours with an ER or PgR content of at least 25 fmol/mg protein were considered positive for ER and PgR, respectively.

ER, oestrogen receptor; GEX, gene expression; HER2, human epidermal growth factor receptor 2; lumA, luminal A; lumB, luminal B; na, not available; PgR, progesterone receptor.

### Subtype-specific genes are often regulated by methylation

**Figure 3:**
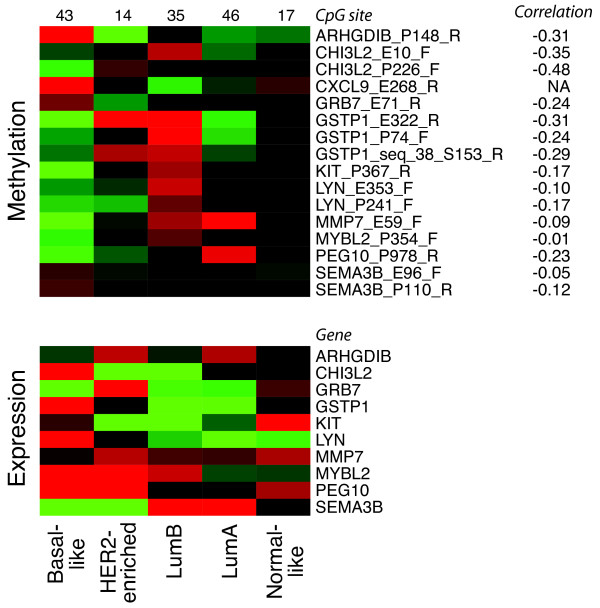
**Heatmaps with average relative methylation and expression levels stratified by subtype**. Subtype expression markers according to Hu *et al. *[14] having subtype-specific methylation are displayed. The number of samples in each subtype (top) and the Pearson correlation between methylation (red, more methylated; green, less methylated) and expression levels (red, high; green, low) are shown. The expression levels correspond well with methylation status. Gene expression data were unavailable for *CXCL9*.

SAM analysis was performed to identify genes differentially methylated for each molecular subtype. Genes that were frequently methylated among lumB tumours were often unmethylated among basal-like tumours, and genes methylated in the basal-like group were more often unmethylated in the lumA group [see Additional File 10]. To investigate whether genes with subtype-specific methylation also were described as gene expression markers for the subtypes, we utilized the gene set that Hu *et al. *generated to build a subtype single sample predictor (SSP) [14]. We had methylation data for 43 of the 301 SSP genes. Of these, we found 11 to have subtype-specific methylation patterns in our SAM analysis and in general these genes showed expression levels that corresponded with methylation status in our data set (Figure 3).

### Breast cancer subtypes and polycomb-regulated genes in ES cells

**Figure 4:**
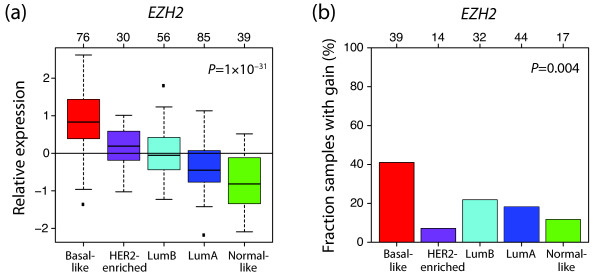
**Relative gene expression levels and genomic gain of *EZH2 *in the different subtypes**. **(a) **Relative expression levels of *EZH2 *across subtypes. Basal-like tumours had the highest expression of *EZH2*. *P*-value was calculated using analysis of variance for all subtypes. **(b) **Fraction of samples with gain of *EZH2*. Gain of this gene is more frequent in basal-like tumours. *P*-value was calculated using Fisher's exact test between basal-like and the other subtypes. The number of tumours in each subtype is shown at the top.

To explore whether genes are silenced in basal-like tumours by other mechanisms than promoter methylation, we utilized gene expression data for 286 primary tumours classified into molecular subtypes to investigate the expression of *EZH2*. We found *EZH2 *to be differently expressed between subtypes (*P *= 1 × 10^-31^, ANOVA; Figure 4a). In particular, basal-like tumours displayed significantly higher expression levels compared with the other subtypes (*P *= 3 × 10^-19^, t-test), consistent with previous observations [36]. Interestingly, *EZH2 *(located on 7q36.1) was frequently gained in basal-like tumours by aCGH (*P *= 0.004, Fisher's exact test; Figure 4b), although no case of high-level amplification was observed. To what extent this can explain the overexpression of *EZH2 *in basal-like tumours remains to be determined.

**Figure 5:**
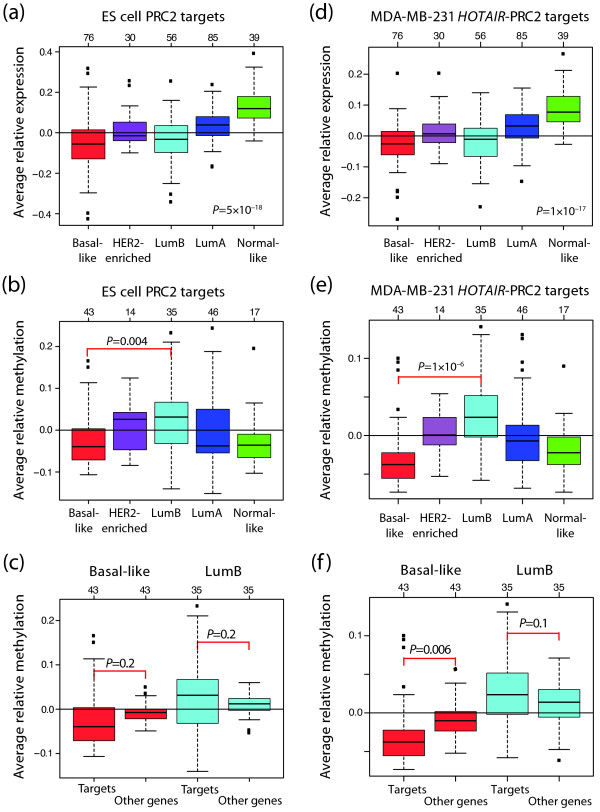
**Relative expression and methylation of PRC2 target genes derived from ES and MDA-MB-231 *HOTAIR *cells**. PRC2 targets identified by Lee *et al. *in **(a to c) **ES cells [28] and Gupta *et al. *[29] by over-expressing *HOTAIR *in **(d to f) **MDA-MB-231 cells, and present in our gene expression data set or methylation panel, respectively, were used. **(a and d) **Average relative expression levels of PRC2 target genes. Basal-like and lumB tumours both have low expression of these genes compared with the other subtypes. *P*-values were calculated using analysis of variance. **(b and e) **Average relative methylation levels of PRC2 target genes. Low methylation levels are found in basal-like tumours while lumB tumours display high levels of methylation of these CpG sites. *P*-values were calculated using t-test between basal-like and lumB tumours. **(c and f) **Average relative methylation levels for PRC2 target genes compared with other genes for basal-like and lumB tumours. *P*-values were calculated using t-test. The number of tumours in each subtype is shown at the top.

To further investigate the role of *EZH2 *in basal-like tumours, we identified 225 PRC2 target genes present in our gene expression data set by using an ES cell PRC2 target gene set identified by Lee *et al. *using ChIP arrays [28]. The average expression levels for these genes stratified by molecular subtype revealed that basal-like and lumB tumours both have low expression of genes that are targets of PRC2 in ES cells (*P *= 5 × 10^-18^, ANOVA; Figure 5a). For this PRC2 target gene set, we identified 134 CpG sites, corresponding to 64 genes, for which we had methylation data. Intriguingly, basal-like tumours have low average relative methylation levels of these CpG sites while lumB tumours display high levels (*P *= 0.004, t-test; Figure 5b). Additionally, there was a tendency towards ES cell PRC2 target genes being more methylated than other genes for lumB tumours, although not significant (*P *= 0.2, t-test), while these genes had a tendency to be less methylated than other genes for basal-like tumours (*P *= 0.2, t-test; Figure 5c).

To investigate the extent to which genes with subtype-specific expression or methylation patterns are also PRC2 targets in ES cells, we investigated three overlaps between gene sets. First, of the 301 SSP genes with subtype-characteristic expression patterns, only four genes (*DUSP4*, *GATA3*, *HOXB6 *and *SFRP1*) were identified by Lee *et al. *as PRC2 targets in ES cells. Second, of 27 genes with strong positive correlation (correlation >0.6) to the gene expression level of oestrogen receptor 1 (*ESR1*) in an expression module for ER status developed by Desmedt *et al. *[37], only three (*ERBB4*, *FBP1 *and *GATA3*) were also in the ES cell PRC2 target gene set. Finally, of the 163 unique genes with methylation patterns associated with the molecular subtypes [see Additional File 9], 15 genes were in the PRC2 target gene set. Hence, although PRC2 targets are differentially methylated across the molecular subtypes, it is clear that many genes with subtype-characteristic expression or methylation in breast tumours are not PRC2 targets in ES cells.

### Subtypes and polycomb-regulated genes in breast cancer cells

To address whether genes under PRC2 control in tumour cells corroborate our findings, we also investigated a polycomb target gene set derived from overexpression of the large intervening non-coding RNA (lincRNA) *HOTAIR *in the ER-negative and basal-like [19] breast cancer cell line MDA-MB-231 [29]. Overexpression of *HOTAIR *in epithelial cells leads to rearrangement of the PRC2 binding pattern towards the one of a less differentiated embryonic fibroblast, and to increased cell invasion and metastatic potential [29]. We had expression data for 288 genes and methylation data for 50 genes (98 CpG sites) in the MDA-MB-231 *HOTAIR*-PRC2 gene set. Using this gene set, we obtained similar results as for the ES cell PRC2 gene set (Figure 5). The relative expression of these genes was significantly different between subtypes (*P *= 1 × 10^-17^, ANOVA), and basal-like and lumB tumours showed relatively low expression of these genes (Figure 5d). High relative methylation in lumB tumours and low in basal-like tumours were also seen for this set of PRC2 targets (*P *= 1 × 10^-6^, t-test; Figure 5e). In this case PRC2 target genes also had a tendency to be more methylated than other genes in lumB tumours (*P *= 0.1, t-test), while being less methylated than other genes in basal-like tumours (*P *= 0.006, t-test; Figure 5f).

**Figure 6:**
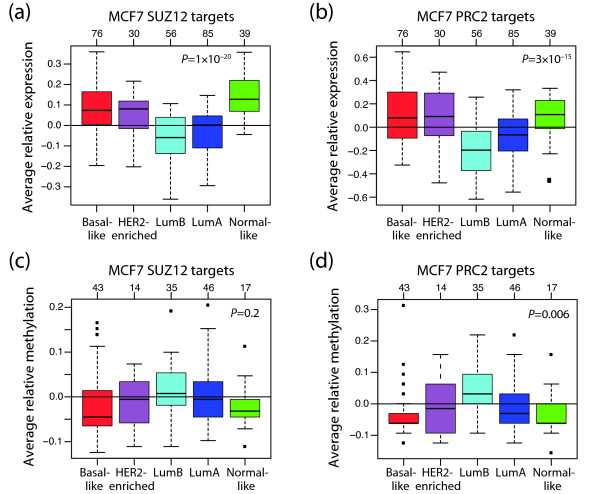
**Relative expression and methylation of SUZ12 and PRC2 target genes derived from MCF7 breast cancer cells**. **(a and c) **SUZ12 targets identified by Squazzo *et al. *[30] and **(b and d) **PRC2 targets identified by Tan *et al. *[31], and present in our gene expression data set or methylation panel, respectively, were used. **(a and b) **Average relative expression of SUZ12 and PRC2 targets, respectively. LumA and especially LumB tumours, have low expression of these genes. *P*-values were calculated using analysis of variance (ANOVA). **(c and d) **Average relative methylation of SUZ12 and PRC2 targets, respectively. Higher methylation levels are found for lumB than lumA tumours. *P*-values were calculated using ANOVA. The number of tumours in each subtype is shown at top.

Finally, we addressed whether luminal breast tumours display a distinct pattern of repressed PRC2 targets. It has been shown that PRC2 binds to promoters in a cell-type specific manner and can be displaced from promoters from one set of genes, while being recruited to another set during lineage specification [8,30]. Squazzo *et al. *have shown that SUZ12 (a member of PRC2) binds to promoters of glycoproteins and immunoglobulin-like proteins in adult MCF7 breast cancer cells, whereas in embryonic cells they bind to genes involved in transcriptional regulation such as homeodomain-containing transcription factors [30]. To investigate this issue, we used two gene sets of polycomb targets derived from the ER-positive and luminal [19] breast cancer cell line MCF7. For the first gene set consisting of targets for SUZ12 [30] (hereafter called MCF7 SUZ12 targets), we had gene expression data for 114 genes and methylation data for 20 genes (38 CpGs). For the second gene set consisting of 44 PRC2 target genes [31] (hereafter called MCF7 PRC2 targets), we had gene expression data for 29 genes and methylation data for 8 genes (16 CpGs). Both lumA and lumB tumours had low relative expression of the genes in these gene sets, while basal-like had high relative expression (*P *= 1 × 10^-20 ^and *P *= 3 × 10^-15^, ANOVA, respectively; Figures 6a and 6b). Interestingly, the genes in these two gene sets tended to be more methylated in lumB than in lumA tumours (Figures 6c and 6d); however, it only reached statistical significance using the MCF7 PRC2 targets (*P *= 0.3 and *P *= 0.02, respectively, t-test). Taken together, these results suggest that unique PRC2 occupation patterns exist for the different subtypes.

## Discussion

In the present study, we used an array-based technology to investigate the methylation status of 807 selected cancer-related genes. By performing unsupervised clustering of 189 breast tumours, we found that basal-like, lumA and lumB tumours have different methylation profiles (Figure 1). On the other hand, tumours of the normal-like and HER2-enriched molecular subtypes did not display distinct methylation profiles. Consistent with our methylation profiling, normal-like tumours do not cluster together based on genomic profiling either [38]. HER2-positive tumours are in general heterogeneous with amplification of the HER2 locus as the common denominator whereas they can be either positive or negative for hormone receptors. Although gene expression profiling has identified a HER2-enriched subtype, it should be noted that HER2-positive tumours are found in all molecular subtypes [22,39], and that expression profiles of HER2-positive tumours are very heterogeneous [23]. Our results add support to the heterogeneous picture of HER2-positive breast cancer, and suggest that *HER2 *amplification does not have a strong characteristic influence on methylation patterns.

Identification of genes with subtype-specific methylation revealed that, for example, *RASSF1 *and *GSTP1 *were specifically methylated in lumB tumours and unmethylated in basal-like tumours. These two genes have previously been shown to be significantly more methylated in ER-positive than in ER-negative tumours [33]. Seven of the genes significantly more methylated in one subtype (*ARHGDIB, GRB7 *and *SEMA3B *in basal-like; *MMP7 *and *PEG10 *in lumA; *GSTP1 *and *CHI3L2 *in lumB) have been shown to have low expression in the corresponding subtype [14]. Moreover, roughly 25% of the genes used for the expression-based SSP molecular subtype classifier [14] and present on our assays were found in our screen for genes with subtype-specific methylation patterns (Figure 3). Taken together, these results suggest that methylation plays a significant role in the different breast tumour phenotypes.

The methylation frequency of genes with methylation patterns associated with the molecular subtypes was significantly higher in lumB tumours than the other subtypes, with basal-like tumours having low methylation frequency (Table 3). The lower degree of methylation observed in basal-like tumours is compatible with their unstable and aberrated genome and is possibly reflected in a reduced transposon silencing [1]. A large difference was also seen between tumours from *BRCA1 *and *BRCA2 *mutation carriers with tumours from *BRCA2 *mutation carriers being significantly more methylated than tumours from *BRCA1 *mutation carriers. This finding emphasises the distinction between hereditary tumours. Using 78 tumours and 11 genes Suijkerbuijk *et al. *[40] found lower methylation frequencies in *BRCA1*-mutated and lymph node-negative tumours than in sporadic and lymph node-positive tumours, respectively. In our larger set of tumours, using more than 800 genes, we could not verify these findings (Table 3). A reason for this discrepancy could be our finding of characteristic methylation patterns for the breast cancer subtypes. As sporadic tumours, lymph node-positive and negative tumours can be found across all subtypes, having a large number of tumours covering all subtypes is essential in comparisons based on clinical variables.

We included normal breast tissue from four breast cancer patients to investigate the difference between methylation frequencies in normal versus cancerous tissue, and found higher frequency in the latter. This is in agreement with previous results by Suijkerbuijk *et al. *[40]. However, the variation in methylation frequency of tumours is large due to differences between molecular subtypes. Interestingly, basal-like tumours showed similar methylation frequencies as the normal tissue samples, whereas luminal tumours showed higher frequencies. It has been suggested that genes having low expression in normal cells undergo *de novo *methylation in tumours [10]. The high methylation frequency in luminal tumours suggests *de novo *methylation. However, direct comparisons of expression and methylation levels in isolated primary luminal cells from normal tissue with levels in luminal tumour tissue would be required to address this further.

An alternative way to epigenetically silence genes is through histone modifications. Trimethylation of H3K27 is a known PRC2-mediated silencing mechanism essential for maintaining stem cells in an undifferentiated state [7]. An analysis of PRC2 target gene sets derived using both ES cells and the basal-like breast tumour cell line MDA-MB-231 revealed low expression of these genes in both basal-like and lumB tumours (Figures 5a and 5d). These results are in accordance with Ben-Porath *et al. *[27] who showed that targets of PRC2 in ES cells had low to moderate expression in both basal-like and lumB tumours. However, analysis of PRC2 targets derived using the luminal breast tumour cell line MCF7, revealed high expression of these genes in basal-like tumours and low in luminal tumours (Figures 6a and 6b), suggesting unique PRC2 target patterns for at least basal-like and luminal tumours. These data are in accordance with Squazzo *et al. *who found that although adult tumour cells (MCF7) and embryonic tumours both have a set of promoters occupied by SUZ12 in common, they also have their own unique SUZ12 occupation pattern [30].

Intriguingly, basal-like tumours displayed low methylation levels of PRC2 target genes in embryonic cells whereas lumB tumours displayed high levels (Figures 5b and 5c). EZH2 is the core member of PRC2, which catalyses the trimethylation of H3K27 [5], and we therefore investigated the expression of this gene in breast cancer. Indeed, we found significantly higher expression in basal-like tumours (Figure 4a) than in the other subtypes. Together, our results suggest that PRC2 target genes in embryonic cells could be silenced through trimethylation of H3K27 in basal-like tumours, whereas in lumB tumours these genes are silenced through promoter methylation. Moreover, polycomb proteins such as EZH2 are involved in stem cell maintenance [6], in line with findings that basal-like breast cancer has a more stem cell-like phenotype [21,41]. Hence, our results suggest it would be valuable to investigate if PRC2 target genes in embryonic cells are silenced by histone modifications in basal-like tumours.

**Figure 7:**
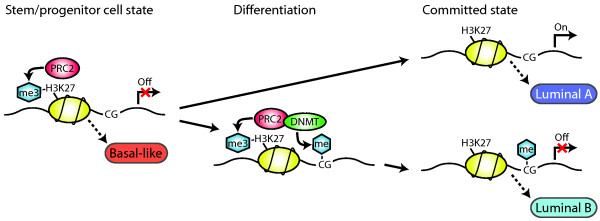
**Potential model for the relations between luminal differentiation and breast cancer subtypes**. PRC2-mediated gene silencing through trimethylation of H3K27 is common in stem/progenitor cells and would be characterised by high EZH2 expression and PRC2 targets having both low expression and unmethylated CpG sites. These characteristics match our findings for basal-like tumours. PRC2 is then displaced (upper path) and PRC2 targets are preferentially activated to promote differentiation. Such a committed cell state would be characterised by low EZH2 expression and PRC2 targets with both high expression and unmethylated promoters. These characteristics match our findings for lumA tumours. In cancer cells, an alternative route for differentiation (lower path), would be to more stably silence PRC2 target genes by promoter methylation. PRC2 associates with DNA methyltransferases (DNMTs) leading to hypermethylation of PRC2 targets. Such a committed cell state would be characterised by low EZH2 expression and PRC2 targets with both low expression and hypermethylated CpG sites. These characteristics match our findings for lumB tumours.

The reason behind the different methylation patterns in the breast cancer subtypes is unknown but could reflect different cellular origins or be driven by mutations in, for example, methyltransferases. Recently, it has been suggested that basal-like tumours originate from an aberrant population of luminal progenitor cells [41]. Our results are compatible with basal-like tumours arising in luminal progenitors in which genes initiating a differentiated luminal cell fate are repressed by PRC2 (Figure 7). During normal differentiation PRC2 is displaced and these PRC2 targets are preferentially activated [42]. Our findings for lumA tumours suggest that they arise in such a differentiated luminal cell (Figure 7). Promoter methylation and histone modifications could silence genes independently [5]. Alternatively, polycomb-mediated methylation of H3K27 could function as a mark of sequences for *de novo *methylation of CpG islands in cancer cells [9,11,43]. In cancer cells, PRC2 has been shown to associate with DNMTs leading to CpG methylation [9], and therefore more permanent repression, of PRC2 target genes. Moreover, a number of studies have shown that genes repressed by PRC2 in ES cells are enriched among genes becoming hypermethylated in cancer [11,34,35,43,44]. We find that our results are compatible with lumB tumours being similar to aberrantly differentiated proliferating luminal cells in which PRC2 targets are methylated (Figure 7). The observed methylation of PRC2 targets is apparently not sufficient to block the differentiation of these cells because lumB tumours share relatively high expression levels of many luminal subtype-specific markers with lumA tumours. Additionally, we found, that some of the genes with subtype-specific expression or co-expression with oestrogen receptor 1 (*ESR1*) in breast cancer were targets of PRC2 in ES cells. An exception is *GATA3*, which is a target of PRC2 in ES cells but highly expressed in luminal tumours. During differentiation PRC2 is relocated to other sets of target genes suggested as a dynamic mechanism to block expression of regulators of alternative cell lineages [8]. We also observed that PRC2 targets in a luminal breast cancer cell line were more methylated in lumB tumours, suggesting that PRC2 targets may become methylated also later in the differentiation of lumB tumours. In addition, overexpression of EZH2 in basal-like tumours could methylate non-histone targets [45] potentially adding further differences between basal-like and lumB tumours.

Somatic mutations in both *EZH2 *and the H3K27 demethylase gene *KDM6A *(*UTX*) have been found in human cancer [46,47]. It may be that somatic alterations in histone methyltransferases contribute to the different methylation patterns for the breast cancer subtypes. For example, *EZH2 *mutations have been found to be frequent in large B-cell lymphomas of germinal-cell origin and suggested to underlie the enhanced methylation at PRC2 targets that have been observed in this cancer type [34,46]. It would be interesting to investigate the mutation status of methyltransferases across molecular subtypes of breast cancer to, for example, explore if methylation of PRC2 targets and the general high degree of methylation in lumB tumours are associated with mutations in such genes. Although Kondo *et al. *found that DNA methylation and H3K27me3 in general do not target the same genes in cancer cell lines, they observed high DNA methylation at H3K27me3 targets in the colon cancer cell line SW48 [5]. Interestingly, SW48 is affected by the CpG island methylator phenotype in which many genes are silenced by methylation [48], similar to our findings for lumB tumours. We have used PRC2 targets in ES and breast cancer cells, and future studies will be needed to address whether PRC2 targets in luminal progenitor or ER-negative cells from normal breast tissue are methylated in lumB tumours. Nevertheless, it should be noted that a significant subset of the genes identified as polycomb targets in ES cells are also targets in breast cancer cells [29]. Moreover, it would be valuable to determine if the selected set of CpGs analysed in this study mirrors a more global promoter methylation pattern.

## Conclusions

Using an array-based platform with more than 800 cancer-related genes we have revealed that the molecular subtypes, especially basal-like, lumA and lumB tumours, harbour specific methylation profiles. Our data add a novel layer of information to the differences between the molecular subtypes and the heterogeneous nature of breast cancer.

## Abbreviations

aCGH: array comparative genomic hybridisation; ANOVA: analysis of variance; ChIP: chromatin immunoprecipitation; DNMT: DNA methyltransferase; EED: embryonic ectoderm development; ER: oestrogen receptor; ES: embryonic stem; ESR1: oestrogen receptor 1; EZH2: enhancer of zeste homolog 2; FDR: false discovery rate; GEO: Gene Expression Omnibus; H3K27me3: trimethylated lysine 27 on histone H3; HER2: human epidermal growth factor receptor 2; lumA: luminal A; lumB: luminal B; MeV: MultiExperiment Viewer; PcG: polycomb group; PCR: polymerase chain reaction; PgR: progesterone receptor; PRC2, polycomb repressive complex 2; SAM: significance analysis of microarrays; SSP: subtype single sample predictor; SUZ12: suppressor of zeste 12 homolog.

## Competing interests

The authors declare that they have no competing interests.

## Authors' contributions

KH, CH, ÅB and MR designed the study. KH performed methylation experiments. KH and MR performed data analyses. KH and JVC selected the patients. HO was responsible for the hereditary tumours. KH, CH, JVC and GJ performed expression arrays. KH, CH, JS and GJ performed aCGH. KH wrote the manuscript together with CH and MR. All authors read and approved the final manuscript.

## Supplementary Material

Additional file 1 **Gene expression data set**. Relative gene expression data for 179 samples and 511 probes. Click here for file

Additional file 2 **Gene expression for PRC2 targets and *EZH2***. Relative gene expression levels for *EZH2 *and average relative expression levels for PRC2 target gene sets for 286 samples. Click here for file

Additional file 3 **Sample annotations**. All annotations used for all 189 tumour samples. Click here for file

Additional file 4 **Methylation data set**. Methylation raw data (β-values) for 1,452 CpGs for 189 tumour samples and four normal breast tissue samples. Click here for file

Additional file 5 **Relative methylation levels**. Stratified and centred methylation data for 1,452 CpGs and 189 breast tumours. Click here for file

Additional file 6 **Hierarchical cluster**. A large version of Figure 1a with CpG sites denoted. Click here for file

Additional file 7 **K-means clusters**. K-means clustering results for K = 2 to 5. Click here for file

Additional file 8 **Correlations**. Correlations between expression and methylation for all 906 CpG site and oligonucleotide probe pairs. Click here for file

Additional file 9 **Differentially methylated CpGs**. List of the 196 significant CpGs after analysis of variance (ANOVA). Click here for file

Additional file 10 **Subtype-specific CpGs**. Significant subtype-specific CpGs after SAM analysis. Click here for file
