# Supplementary material for: Molecular subtypes of breast cancer are associated with characteristic DNA methylation patterns
Source: Breast Cancer Res. 2010 Jun 18;12(3):R36. doi: 10.1186/bcr2590 (PMC2917031; doi:10.1186/bcr2590)
Supplement: Additional file 6 — Hierarchical cluster. A large version of Figure 1a with CpG sites denoted. [file bcr2590-S6.PDF]

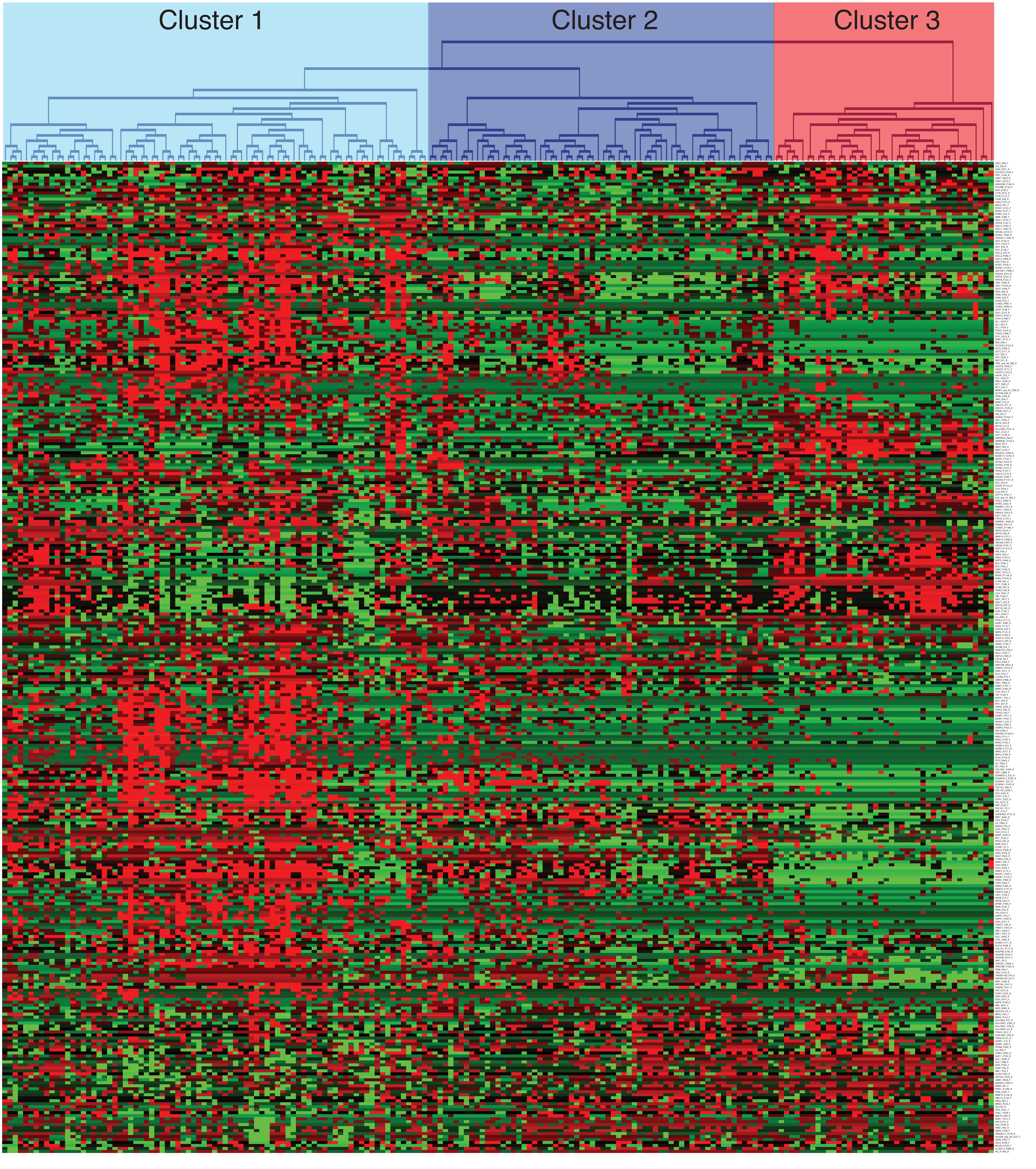
Cluster 1
Cluster 2
Cluster 3
