# Supplementary figures and images for: Molecular subtypes of breast cancer are associated with characteristic DNA methylation patterns
Source: Breast Cancer Res. 2010 Jun 18;12(3):R36. doi: 10.1186/bcr2590 (PMC2917031; doi:10.1186/bcr2590)

K=2

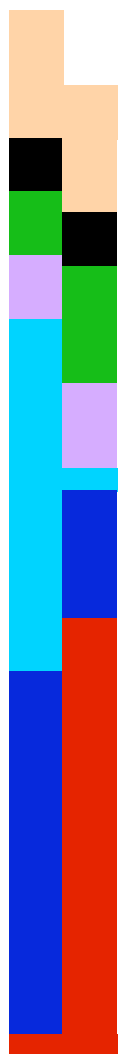

K=3

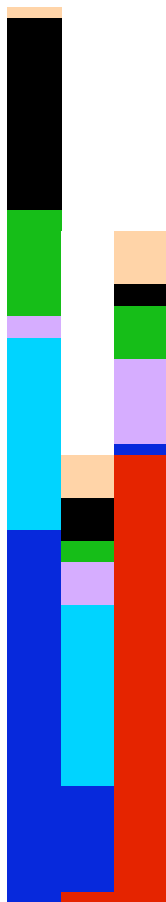

K=4

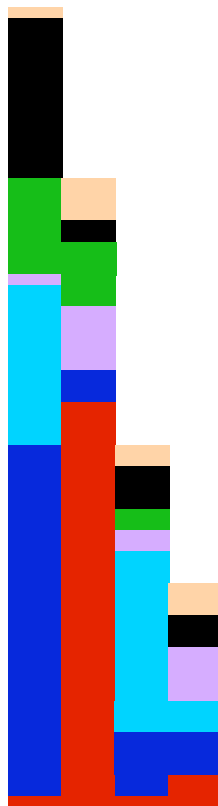

K=5

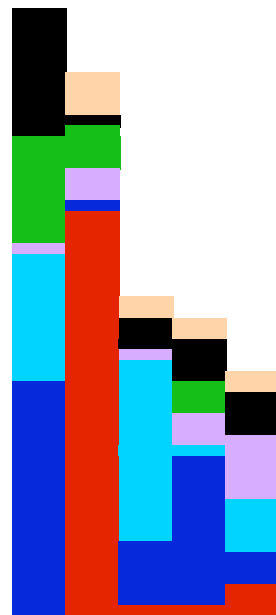

|                |    |    |
|----------------|----|----|
| Basal-like     | 2  | 41 |
| LumA           | 34 | 12 |
| LumB           | 33 | 2  |
| HER2-enriched  | 6  | 8  |
| Normal-like    | 6  | 11 |
| Non-classified | 12 | 12 |
| Non-GEX        | 5  | 5  |
| Total          | 98 | 91 |

|    |    |    |
|----|----|----|
| 0  | 1  | 42 |
| 35 | 10 | 1  |
| 18 | 17 | 0  |
| 2  | 4  | 8  |
| 10 | 2  | 5  |
| 18 | 4  | 2  |
| 1  | 4  | 5  |
| 84 | 42 | 63 |

|    |    |    |    |
|----|----|----|----|
| 1  | 38 | 1  | 3  |
| 33 | 3  | 6  | 4  |
| 15 | 0  | 17 | 3  |
| 1  | 6  | 2  | 5  |
| 9  | 6  | 2  | 0  |
| 15 | 2  | 4  | 3  |
| 1  | 4  | 2  | 3  |
| 75 | 59 | 34 | 21 |

|    |    |    |    |    |
|----|----|----|----|----|
| 0  | 38 | 1  | 1  | 3  |
| 22 | 1  | 6  | 14 | 3  |
| 12 | 0  | 17 | 1  | 5  |
| 1  | 3  | 1  | 3  | 6  |
| 10 | 4  | 0  | 3  | 0  |
| 12 | 1  | 3  | 4  | 4  |
| 0  | 4  | 2  | 2  | 2  |
| 57 | 51 | 30 | 28 | 23 |

Supplement: Additional file 7 — K-means clusters. K-means clustering results for K = 2 to 5. [file bcr2590-S7.PDF]
